# Editorial: Molecular advances and applications of machine learning in understanding autism and comorbid psychiatric disorders

**DOI:** 10.3389/fnmol.2023.1277814

**Published:** 2023-08-31

**Authors:** Salam Salloum-Asfar

**Affiliations:** Neurological Disorders Research Center, Qatar Biomedical Research Institute, Hamad Bin Khalifa University, Qatar Foundation, Doha, Qatar

**Keywords:** autism, neurodevelopmental disorders, molecular factors, genomic, transcriptome, artificial intelligence, modeling

Welcome to this Research Topic titled “*Molecular Advances and Applications of Machine Learning in Understanding Autism and Comorbid Psychiatric Disorders*.” In assembling this Research Topic, I express my gratitude to the dedicated reviewers who thoughtfully evaluated submissions, and to the authors who entrusted us with their valuable research, allowing us to enhance and refine their work.

This editorial encapsulates 15 meticulously curated articles, contributing to the profound exploration of neurodevelopmental disorders (NDDs), with a distinct focus on autism spectrum disorder (ASD) and its intricate interplay with comorbid psychiatric conditions. The contributions cast light on the intricate etiology and molecular mechanisms underlying these complex disorders. This Research Topic examines myriad dimensions, encompassing various facets, such as genetic predisposition, dynamic gene expression, signal transduction pathways, compensatory mechanisms, and neural network organization. A summary of the 15 accepted articles is provided below in eight categories:

## 1. Gut microbiota, depression, and neurodevelopmental dysfunction

The study from Liu et al. uncovers the impact of gut microbiota-dysbiosis on hippocampal gene regulation, elucidating the significant role of molecular dysregulation in neurodevelopmental dysfunction.

## 2. Cerebellar dysfunction and autism

Yang et al. meticulously examine the implications of SCN8A gene knockout in cerebellar Purkinje cells. The research unveils compromised social interaction, motor learning, reversal learning, and cerebellar degeneration, with mutations in the SCN8A gene linked to epilepsy, intellectual disability, and ASD.

## 3. Sensory processing and behavior problems in ASD

The study by Alateyat et al. employs machine learning models to predict behavior outcomes based on sensory profile scores, shedding light on the intricate interplay between sensory processing abilities and behavioral patterns in ASD.

## 4. Brain laterality, AI, and MRI neuroimaging in ASD detection

Keeratitanont et al. investigate brain laterality by F-18 fluorodeoxyglucose positron emission computed tomography (PET/CT) among high-functioning ASD individuals. The study uncovers altered glucose metabolism and lateralization indices, hinting at potential left laterality aberrations as contributory factors to ASD. Moreover, Moridian et al. review artificial intelligence (AI) and MRI neuroimaging for automatic ASD detection. Various AI methods, including machine learning (ML) and deep learning (DL), are assessed for their potential in accurate and efficient ASD diagnosis.

## 5. Genomic, transcriptomic factors in ASD, and genetic similarities between ASD and comorbid brain disorders

Multiple studies provide valuable insights into genomic and transcriptomic factors in ASD and their shared genetic traits with comorbid brain disorders. Chen et al. investigated the role of the NECAB2 gene, uncovering its impact on psychomotor and social behavior via mGluR1 signaling modulation. Krgovic et al. employed whole exome sequencing to spotlight ultrarare variants in ASD-associated genes, revealing their interconnectedness with various NDDs. Vilela et al. genetic similarity disease network study unveiled shared genetics between ASD and comorbid brain disorders, revealing novel insights into shared biological pathways and underlying mechanisms.

Mahmoud et al. comprehensively explored the genomic landscape of 1p13.3, identifying autosomal dominant candidate genes and emphasizing the role of small CNVs in shaping clinical outcomes. Yoo Y. E. et al. studied transcriptomic changes in Shank2-mutant mice, revealing distinct patterns across brain regions, gene dosages, and ages, shedding light on the dynamic interplay between Shank2 mutations and brain region-specific transcriptomic alterations. George-Hyslop et al. reviewed CNTNAP2's multifaceted role in neurodevelopmental disorders and human cerebral cortex evolution, highlighting its association with conditions like ASD and SLI. Shen et al. provided an overview of Gadd45b's preclinical and clinical effects, hypothesized mechanisms of action, and its role in various neurological disorders. Yoo T. et al. investigated transcriptomic variations in Shank3-mutant mice, revealing opposing and similar profiles to ASD across different ages, brain regions, and gene dosages. This study highlighted the intricate interplay of age, brain region, and gene dosage in shaping transcriptomic changes.

## 6. Homocysteine metabolism and ASD

An investigation by Li et al. into the interplay between serum homocysteine, folate, and vitamin B12 levels, assessing their correlation with ASD clinical manifestations and severity, underscoring the pertinence of homocysteine metabolism dysregulation in the context of ASD.

## 7. The role of MAP2 in neurodevelopmental disorders

A hypothesis and theory article by DeGiosio et al. postulates the potential pathogenic functions of the microtubule-associated protein 2 (MAP2) in NDDs. While traditionally recognized as a somatodendritic marker, its intricate influence on microtubule dynamics and neurite outgrowth is discussed, highlighting its relevance in various neurodegenerative and neuropsychiatric conditions.

Overall, this compendium of research articles synthesizes a rich tapestry of insights into the intricate molecular fabric of ASD and comorbid psychiatric disorders. By comprehending these underlying mechanisms, the prospects for early intervention and improved outcomes for affected individuals are significantly augmented. It is my sincere hope that this compilation serves as a pivotal steppingstone toward more precise diagnostics, individualized treatments, and enhanced therapeutic interventions in the realm of neurodevelopmental disorders.

## Author contributions

SS-A: Investigation, Supervision, Writing—original draft, Writing—review and editing.

